# EndoG Links Bnip3-Induced Mitochondrial Damage and Caspase-Independent DNA Fragmentation in Ischemic Cardiomyocytes

**DOI:** 10.1371/journal.pone.0017998

**Published:** 2011-03-17

**Authors:** Jisheng Zhang, Junmei Ye, Albert Altafaj, Maria Cardona, Núria Bahi, Marta Llovera, Xavier Cañas, Stuart A. Cook, Joan X. Comella, Daniel Sanchis

**Affiliations:** 1 Institut de Recerca Biomèdica de Lleida (IRBLLEIDA), Universitat de Lleida, Lleida, Spain; 2 Parc Científic de Barcelona, Barcelona, Spain; 3 Medical Research Council Clinical Sciences Centre, Imperial College, Hammersmith Hospital Campus, London, United Kingdom; 4 Ciberned, Institut de Neurociències, Hospital Vall d'Hebró, UAB, Barcelona, Spain; Florida International University, United States of America

## Abstract

Mitochondrial dysfunction, caspase activation and caspase-dependent DNA fragmentation are involved in cell damage in many tissues. However, differentiated cardiomyocytes repress the expression of the canonical apoptotic pathway and their death during ischemia is caspase-independent. The atypical BH3-only protein Bnip3 is involved in the process leading to caspase-independent DNA fragmentation in cardiomyocytes. However, the pathway by which DNA degradation ensues following Bnip3 activation is not resolved. To identify the mechanism involved, we analyzed the interdependence of Bnip3, Nix and EndoG in mitochondrial damage and DNA fragmentation during experimental ischemia in neonatal rat ventricular cardiomyocytes. Our results show that the expression of EndoG and Bnip3 increases in the heart throughout development, while the caspase-dependent machinery is silenced. TUNEL-positive DNA damage, which depends on caspase activity in other cells, is caspase-independent in ischemic cardiomyocytes and ischemia-induced DNA high and low molecular weight fragmentation is blocked by repressing EndoG expression. Ischemia-induced EndoG translocation and DNA degradation are prevented by silencing the expression of Bnip3, but not Nix, or by overexpressing Bcl-x_L_. These data establish a link between Bnip3 and EndoG-dependent, TUNEL-positive, DNA fragmentation in ischemic cardiomyocytes in the absence of caspases, defining an alternative cell death pathway in postmitotic cells.

## Introduction

Caspase activation has been involved in the pathophysiology of the cell death processes in terminally differentiated cells including neurons and cardiomyocytes. However, recent results show that proteins in the canonical apoptotic signaling pathway are not detectable in postmitotic cells [1], [2] and the role of caspases in some diseases such as ischemia remains controversial [3], [4][. In this context, elucidation of the mechanisms mediating caspase-independent cell damage could be relevant for understanding alternative pathways to caspase-dependent cell death.

Activation of the apoptotic mitochondrial pathway in many cell types includes interaction of a BH3-only protein with components of the outer mitochondrial membrane (OMM), mitochondrial dysfunction, apoptosome assembly, caspase activation and DNA processing by Caspase-Activated DNAse (CAD/DFF40) [5]. Bnip3 and Nix form an atypical subfamily of the BH3-only proapoptotic proteins related to Bcl-2 [6]. Their expression was described to be activated by hypoxia, involving Hypoxia Inducible Factor-1 (HIF-1)-dependent transcription [7], [8]. Surprisingly, Bnip3 was shown to be abundant in the adult heart in normoxia [9]. Bnip3 has been involved in hypoxia/acidosis-induced DNA damage in cardiomyocytes [10]. Increased resistance to hypoxia-induced cell death of pancreatic and colorectal cancer cells deficient for Bnip3 further supports the relevance of Bnip3 during hypoxia-induced death [11], [12]. Integration of Bnip3 in the OMM leads to mitochondrial damage [13]–[15], including opening of the mitochondrial permeability transition pore [16]. Deficiency in Bnip3 in vivo has been shown to improve heart function after ischemia/reperfusion although the mechanisms involved are not clear [17]. Finally, Bnip3 induces caspase-independent nuclear DNA degradation [10], [16]. This fact has been described as surprising [16], unusual [7], [10], interesting [18] and paradoxical [19] in cardiomyocytes but remains unexplored.

Endonuclease-G (EndoG) is a mitochondrial protein with DNase/RNase activity [20], which has been recently shown to be involved in apoptotic DNA degradation [21], [22]. EndoG apoptotic activity requires its release from mitochondria, and occurs in the absence of caspase activation [21]. Although Li and Wang demonstrated that EndoG activity is relevant only in caspase-independent cell damage, caspase-dependent paradigms were used for the phenotypic characterization of EndoG-deficient mice, which were the basis for discarding a relevant role of EndoG in apoptosis [23], [24]. We have recently reported that EndoG is important for DNA degradation in cardiomyocytes, where the caspase-dependent pathway is repressed during differentiation and is not re-expressed during experimental ischemia [25]. However, the pathway leading to EndoG-dependent DNA damage remains debated [26].

We hypothesized that EndoG could be the executor of caspase-independent DNA processing in the Bnip3-mediated cardiomyocyte death pathway. The results presented here support this hypothesis and help to clarify other functional aspects dealing with ischemia-induced cell death, such as the role of endogenous Bnip3 in triggering translocation of mitochondrial apoptotic regulators in the absence of caspase activity, and the caspase-independent nature of the TUNEL labeling in damaged cardiomyocytes.

## Results

### EndoG and Bnip3 are expressed during development and are abundant in the adult heart, contrary to the caspase-dependent protein network

**Figure 1:**
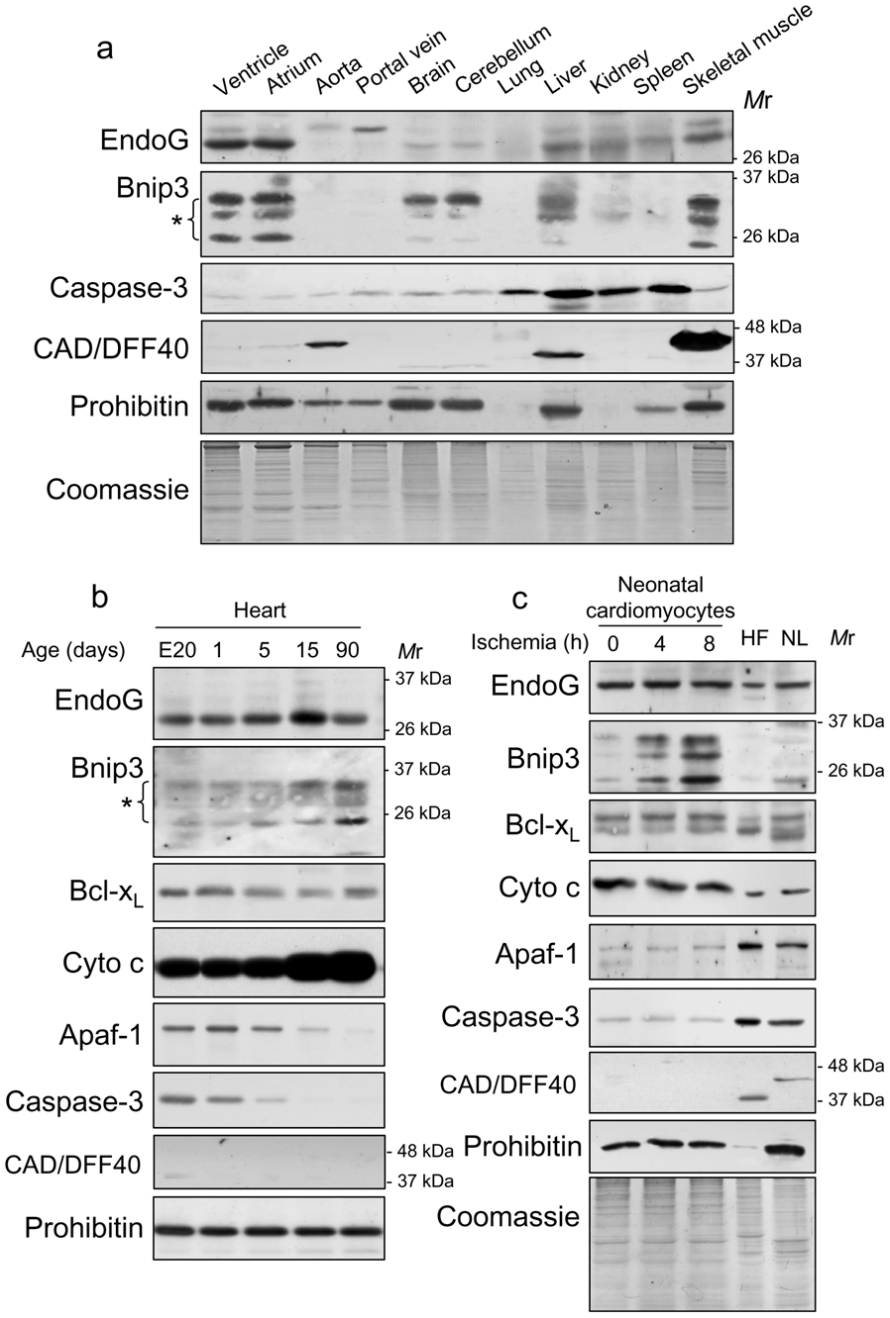
EndoG and Bnip3 are most highly expressed in rat heart, and are located to cardiac myocytes thorough development and during ischemia. a) Western blot analysis of EndoG, using a new anti-EndoG antibody (AntibodyBcn, BCN4778) and Bnip3 protein expression in total protein extracts from different adult rat tissues. EndoG is detected as a single band (∼27 kDa) and Bnip3 is detected as a group of bands around the 26 kDa mark (asterisk), in agreement with the information provided by the manufacturer. Both proteins are expressed mainly in the heart and skeletal muscle. Detection of EndoG and Bnip3 was performed in two different sets of independent samples with similar results. b) Western blot detection of EndoG, Bnip3 and proteins involved in caspase-dependent cell death in total protein extracts of ventricles from rats of different ages ranging from embryonic day 20 to adulthood. A representative image is shown from three independent experiments. c) Neonatal cardiomyocytes were treated with ischemia and the abundance of EndoG, Bnip3 and proteins involved in the caspase-dependent pathway was analyzed by Western blot in extracts obtained at different time points. Primary heart fibroblasts and neonatal liver samples were added for comparison. A representative image is shown from three independent experiments.

We have previously shown that many key regulators of the caspase-dependent cell death signaling cascade are abundant in the embryonic heart but their expression is downregulated during development and these proteins are not re-expressed during experimental ischemia or ischemia-reoxygenation in vitro [2]. Our previous results and data presented here show that experimental ischemia does not induce caspase activation in primary rat cardiomyocytes and that ischemia-induced DNA damage in these cells depends on EndoG (Figures S1 and S2 and ref. [25]). Here we extend the knowledge about EndoG expression. We produced an EndoG-specific antibody (AntibodyBcn, BCN4778) because in our hands the majority of commercial antibodies detected an unspecific band (Figure S3). Our data show that EndoG is most abundant in the heart, skeletal muscle and liver in the adult rat (Figure 1a). In the cardiac ventricle, EndoG expression increases during heart development contrary to Apaf-1, Caspase-3 and CAD, which are key regulators of the caspase-dependent death pathway (Figure 1b). Bnip3 protein has been shown to be abundant only during hypoxia in several cell types [8], [12] but recently it has been reported to be expressed in the adult heart in normoxia [9]. Using a different anti-Bnip3 antibody (Cell Signaling, 3769), we have found that Bnip3 protein is mainly expressed in the heart, brain and skeletal muscle in the adult rat (Figure 1a) and its abundance increases in the heart during development (Figure 1b) and during hypoxia in neonatal myocytes (Figure 1c), contrary to the genes regulating the caspase-dependent pathway that are highly expressed in other cell types and are not re-expressed in myocytes during ischemia (Figure 1c). Anti-apoptotic Bcl-x_L_ is the only Bcl-2-related protein abundant in the adult heart (ref. [2] and Figure 1b). Thus, our results suggest that in the differentiated myocardium and in ischemic myocytes EndoG, Bnip3 and Bcl-x_L_ are abundant whereas the expression of the caspase-dependent cell death machinery is silenced and is not re-expressed during ischemia.

### Bnip3 and EndoG trigger caspase-independent, TUNEL positive DNA fragmentation in ischemic cardiomyocytes, which is a caspase-independent paradigm

**Figure 2:**
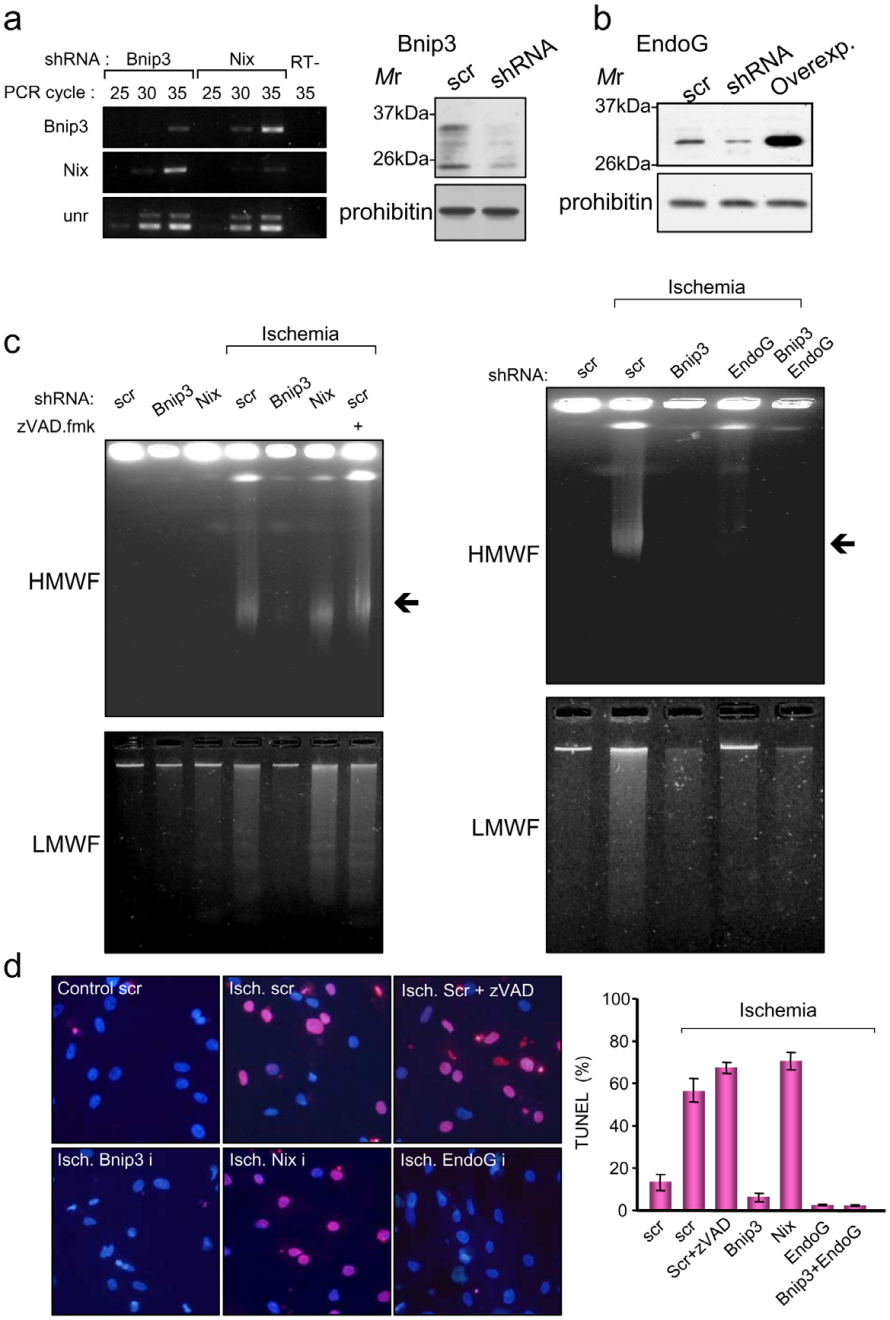
Bnip3 and EndoG trigger caspase-independent TUNEL positive DNA fragmentation in ischemic cardiomyocytes. a) Efficiency of Bnip3 and Nix shRNA-mediated knock down was analyzed by RT-PCR and / or Western blot from postnatal cardiomyocyte total RNA or protein extracted at day 4 post-transduction. b) Efficiency of EndoG shRNA-mediated knock down and specificity of the new anti-EndoG antibody was analyzed by Western blot in the same conditions than in a. c) Agarose gel electrophoresis of DNA extracts of cardiomyocytes cultured in normal conditions or after 16 hours of experimental ischemia. Cardiomyocytes were transduced 5 days before ischemia with constructs for the silencing of Bnip3, Nix or EndoG genes. A sample from scrambled-transduced cardiomyocytes treated with ischemia in the presence of 100 µM pan-caspase inhibitor z-VAD-fmk was added to show that DNA fragmentation was caspase-independent in this paradigm. HMWF: High molecular weight DNA fragmentation detected by pulse-field electrophoresis as reported in the Materials and Methods section. LMWF: Low molecular weight DNA fragmentation detected by conventional agarose gel electrophoresis from the same cell extracts. d) DNA integrity was measured by the TUNEL assay following the same treatments as in B. TUNEL staining was detected as a red fluorescent signal, which, in combination with blue Hoechst nuclear staining produced a pink hue. Gel images are representative of three independent experiments. TUNEL-positive nuclei (pink) are expressed as mean vs. total nuclei (blue+pink) from three independent experiments counted in duplicates. Scr: scrambled. Error bars are s.e.m.

Bnip3 has been involved in chromatin condensation [13], and in ischemia-induced cardiomyocyte DNA low molecular weight (LMW) fragmentation [10]. Although the Bnip3-related protein Nix also has a pro-apoptotic role [6], [17] and its expression is up-regulated during hypoxia in several cell lines [7], [8], its involvement in hypoxia-induced cell death is less clear [27]. We developed lentiviral-based small hairpin RNA interference systems (shRNA) for silencing efficiently Bnip3 and Nix expression in primary postnatal cardiomyocytes (Figure S2, Figure 2, a and b). Silencing Bnip3 expression, but not that of Nix, blunted high molecular weight (HMW) and LMW DNA degradation during experimental ischemia (Figure 2c). The effect of Bnip3 silencing on DNA integrity during ischemia was similar to this of repressing EndoG (Figure 2c, right panel). Both EndoG and Bnip3, but not Nix, were essential for inducing TUNEL-positive DNA fragmentation in experimental ischemia, which did not involve caspase activity (Figure 2d). Thus, caspase-independent EndoG activity was involved in the production of DNA -3'OH single strand nicks detected by the TUNEL assay, which has been routinely used as a measure of Caspase-Associated DNase (CAD) activity and, hence, caspase activation in cardiomyocytes. Survival of ischemic cardiomyocytes, measured in control (100% survival) and ischemia by the trypan blue exclusion assay, was not affected by silencing Bnip3 expression (67.0±9.74% vs. 62.7±8.13% scrambled-transduced vs. shBnip3-transduced cardiomyocytes, n = 3). These data improve former knowledge by showing that Bnip3, but not Nix, is involved in ischemia-induced DNA high as well as low molecular weight fragmentation, and that ischemic DNA damage detected by the TUNEL assay proceeds in the absence of caspase activation in cardiomyocytes.

### BNIP3 controls EndoG release from cardiac mitochondria and Bcl-x_L_ can block EndoG translocation and caspase-independent DNA damage

**Figure 3:**
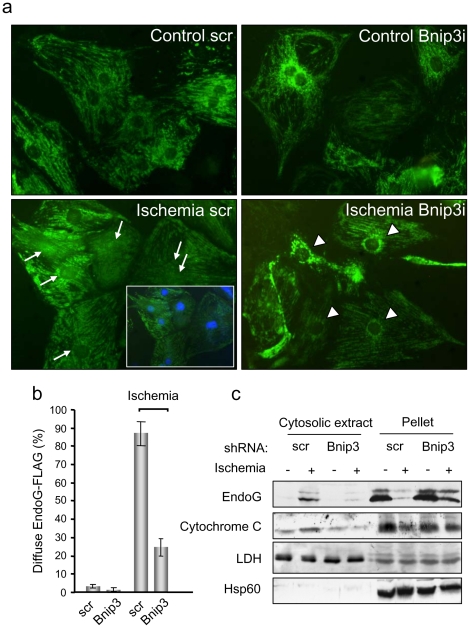
BNIP3 controls EndoG release from cardiac mitochondria during ischemia. a) Immunofluorescence of EndoG-FLAG in overexpressing neonatal cardiomyocytes cultured in control (control) and ischemic (ischemia) conditions for 12 hours. Bnip3i: cardiomyocytes with silenced expression of Bnip3 by shRNA. Scr: scrambled. Arrows: cardiomyocytes where EndoG-FLAG expression includes the nucleus. A merged image including Hoechst nuclear staining is included for ischemic cardiomyocytes to localize the nuclei. Arrowheads: ischemic cardiomyocytes where EndoG-FLAG expression excludes the nucleus. Similar results were obtained in three independent experiments. b) Quantification of the experiments described in a). The graph shows the percentage of cardiomyocytes with diffuse pattern of the EndoG-FLAG staining in control and ischemic cultures of scrambled-transduced cells (scr) and cells transduced with viruses for Bnip3 silencing (Bnip3), counted in three independent experiments. Error bars are s.e.m. c) Endogenous EndoG release was assessed by Western Blot in cytosolic extracts from control cardiomyocytes (scr) and cardiomyocytes expressing low levels of Bnip3 (achieved by shRNA-driven knockdown) in standard and ischemic conditions. Lactate dehydrogenase (LDH) expression was checked as a cytosolic marker and Hsp60 expression was assessed as a mitochondrial membrane marker. The image shows representative results from 3 independent experiments.

Experimental ischemia induced the release of EndoG-FLAG from cardiomyocyte mitochondria as shown by the diffuse pattern of expression in ischemic cardiomyocytes treated with 12 hours of experimental ischemia and fixed with 4% PFA, where staining also included cell nuclei (Figure 3a and b). Silencing Bnip3 expression by shRNA contributed to maintain EndoG-FLAG normal localization, excluding the nucleus (Figure 3a and b). Western blot analysis of EndoG-FLAG expression in cytosolic extracts of cardiomyocytes in the same conditions confirmed that Bnip3 silencing prevented EndoG-FLAG release from mitochondria (Figure 3c).

**Figure 4:**
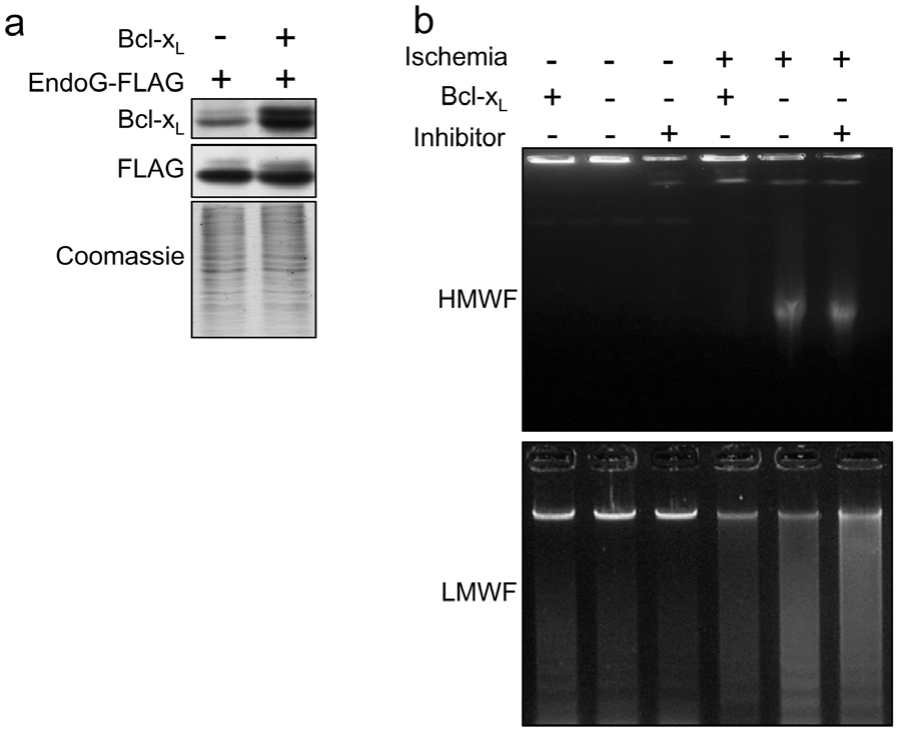
Bcl-x_L_ can block caspase-independent DNA damage in ischemic cardiomyocytes. a) Assessing overexpression of Bcl-x_L_ in normal cardiomyocytes and simultaneous EndoG-FLAG overexpression. b) Agarose gel electrophoresis of DNA extracts of cardiomyocytes cultured in normal conditions or after 6 hours of experimental ischemia. Cardiomyocytes were transduced 5 days before ischemia with lentivirus for overexpression of Bcl-x_L_ or empty viruses. HMWF and LMWF: high and low molecular weight DNA fragmentation, respectively. Similar results were obtained in three independent assays.

**Figure 5:**
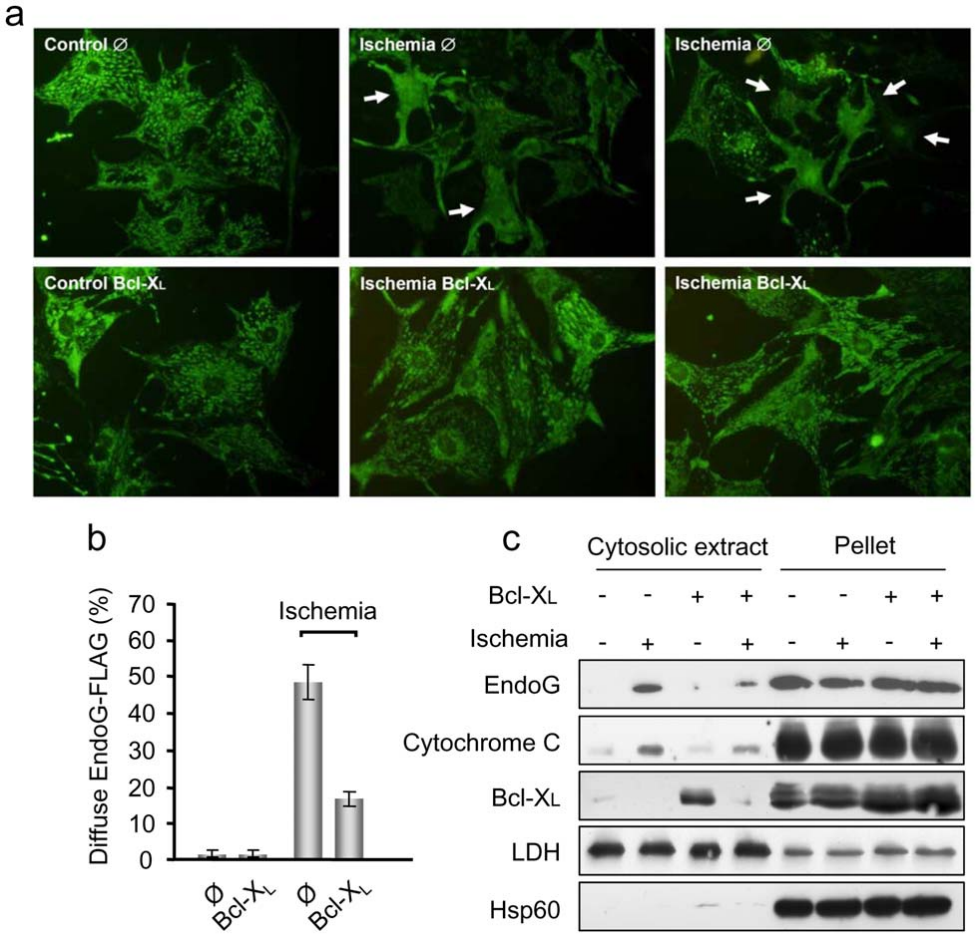
Bcl-x_L_ blocks EndoG translocation in ischemic cardiomyocytes. a) Immunofluorescence of EndoG-FLAG in control and ischemic (6 hours) cardiomyocytes transduced previously with viruses inducing EndoG-FLAG overexpression and empty viral particles (Ø) or particles inducing Bcl-x_L_ overexpression. Cells were fixed with paraformaldehyde. This procedure allows detection of EndoG-FLAG, even when it is released from mitochondria, but not the mitochondrial marker Hsp60 (see the results section and Supplementary Figure 3). b) Quantification of the experiments described in a). The graph shows the percentage of cardiomyocytes with diffuse pattern of the EndoG-FLAG staining in control and ischemic cultures of empty virus-transduced cells (Ø) and cells transduced with viruses for Bcl-x_L_ overexpression (Bcl-x_L_), counted in three independent experiments. Error bars are s.e.m. c) EndoG-FLAG release was assessed by Western Blot in cytosolic extracts from control cardiomyocytes (transduced with EndoG-FLAG and empty viruses) and cardiomyocytes overexpressing Bcl-x_L_ (transduced with EndoG-FLAG and Bcl-x_L_ viruses), in standard and ischemic conditions. Expression of Bcl-x_L_ was assessed to confirm its increased expression in cardiomyocytes transduced with Bcl-x_L_. Lactate dehydrogenase (LDH) expression was checked as a cytosolic marker and Hsp60 expression was assessed as a mitochondrial membrane marker. The image shows representative results from 3 independent experiments.

Bcl-x_L_ overexpression has been shown to protect cardiac mitochondria during ischemia and to reduce DNA fragmentation detected by TUNEL in a Langendorf perfusion model [28]. Thus, we assessed whether Bcl-x_L_, which is expressed in the differentiated myocardium (as shown in Figure 1b), was efficient in blocking EndoG nuclease activity. Bcl-x_L_ overexpression was induced in cultured cardiomyocytes before ischemia (Figure 4a) and DNA integrity was analyzed by agarose gel electrophoresis in control and ischemic conditions. The results suggest that Bcl-x_L_ overexpression blocks DNA fragmentation during ischemia for 6 hours (Figure 4b) in the absence of detectable executioner caspase activation (Figure S1). In order to know whether Bcl-x_L_ prevented EndoG translocation during ischemia, EndoG expression was analyzed by immunofluorescence in control and ischemic cardiomyocyte cultures, which were also overexpressing EndoG-FLAG. Fixation with PFA allowed detection of EndoG-FLAG even when released from mitochondria. In empty virus-transduced ischemic cardiomyocytes the pattern of EndoG-FLAG expression was diffuse (Figure 5a), suggesting translocation, which was significantly prevented in cardiomyocytes overexpressing Bcl-x_L_ (Figure 5a and b). Western blot analysis of EndoG-FLAG expression in cytosolic extracts of cardiomyocytes in the same conditions confirmed that Bcl-x_L_ overexpression prevented EndoG-FLAG release from mitochondria (Figure 5c). To complete these results, we fixed other cultures from the same experiment with methanol. This procedure allowed co-staining of EndoG-FLAG and the mitochondrial marker Hsp60, demonstrating co-localization (merged image) in control conditions (Figure S4). However, in ischemic cardiomyocytes, the FLAG staining was lost in absence of Bcl-x_L_ overexpression, probably due to wash out of the released EndoG-FLAG during the immunofluorescence procedure because methanol dissolves membrane lipids. These results demonstrate that EndoG-FLAG localizes to mitochondria and is released during ischemia, and that Bcl-x_L_ overexpression can block this event by protecting mitochondrial integrity. Taken together, our findings showed that Bcl-x_L_ efficiently blocked translocation of overexpressed EndoG and prevented caspase-independent DNA damage in ischemic cardiomyocytes.

## Discussion

The events leading to EndoG-mediated DNA fragmentation and the pathway mediating Bnip3-induced DNA damage are not well understood. Here we show that EndoG and Bnip3 form a protein network involved in caspase-independent DNA fragmentation in differentiated cardiomyocytes during ischemia. Our results suggest that ischemia-induced TUNEL-positive DNA damage in cardiomyocytes in vitro is triggered by EndoG and depends on EndoG release from mitochondria induced by Bnip3 in a way that can be partially blocked by Bcl-x_L_, without the involvement of caspases.

The actual relevance of EndoG activity is still a mater of debate, with papers supporting it in yeast [29], cardiomyocytes [25] and hepatocytes [30] and papers suggesting a minor role of this nuclease in other cell types [23], [24]. Our results complement the knowledge about the relevance of EndoG by showing that EndoG activity depends on Bnip3 without the involvement of caspases in cardiomyocytes. Indeed, EndoG was initially reported to degrade nuclear DNA when caspases were inhibited [21] and has been evidenced in caspase-independent paradigms [25], [29], [30]. Interestingly, the experiments discarding a relevant role of EndoG where performed in caspase-dependent paradigms such as splenocytes [23] and mouse embryonic fibroblasts [24], were caspases and CAD/DFF40 play a major role in DNA degradation.

There is also a certain degree of disagreement about the contribution of the canonical caspase-dependent apoptotic pathway to ischemia-induced cell death, which was initially suggested in cardiomyocytes by Tanaka and co-workers [31]. The extrinsic, death receptor-dependent, apoptotic cascade ([32], and others) and the mitochondria-dependent pathway of apoptosis [33] have been suggested to contribute to cardiomyocyte death during ischemia or ischemia/reperfusion. However, simultaneous reports showed that myocyte damage and death in these and other contexts were unrelated to caspases [3], [4], [10], [25], [34]. The lack of caspase activity in ischemic cardiomyocytes is in agreement with previous findings showing that expression of the apoptotic genes is silenced during cardiomyocyte differentiation [2], [25]. Using different approaches, it has been shown that Bcl-2 and Bcl-x_L_ blunt ischemia-induced cardiomyocyte damage [28], [35], [36], also suggesting the involvement of the caspase-dependent pathway triggered by Cytochrome c translocation. The use of the TUNEL assay and agarose-gel electrophoresis to assess DNA fragmentation as reliable methods for detecting apoptotic myocytes in human samples contributed to the knowledge about the role of cell death in heart failure but unwittingly biased the interpretation of the mechanisms involved in this event [37], [38] and others.

Although many excellent works demonstrated the essential role of Bnip3 in DNA damage in myocytes and other cell types [7], [16], [18], the nuclease executing Bnip3-dependent DNA damage in cardiomyocytes has not been identified so far. Here we show that endogenous Bnip3 induces EndoG release and caspase-independent EndoG-dependent DNA fragmentation, supporting that EndoG executes DNA damage triggered by Bnip3. Our results are in agreement with two recent reports showing the association between Bnip3 and EndoG activity in cortical neurons during long exposure to hypoxia and in hippocampal neurons during reoxygenation although in those cells the process appeared to be much slower than in cardiomyocytes [39], [40]. We also extend the knowledge about the role of Bnip3 in DNA damage by showing for the first time that high molecular weight DNA fragmentation also depends on Bnip3 activity. In addition, although cell damage induced by Bnip3 depends on Bax and Bak activation and induce caspase activation in caspase-dependent cellular models such as embryonic fibroblasts (MEF) and HL-1 immortalized embryonic atrial myocytes [41], our results give support to a role of Bnip3 unrelated to caspases in differentiated cardiomyocytes as previously shown by others [10] and also suggest a caspase-independent mechanism for the known protective role of overexpressed Bcl-x_L_ in the heart [28]. The shorter time of protection from DNA damage achieved by Bcl-x_L_ overexpression compared to Bnip3 silencing in our paradigm could be explained by Bnip3 increased expression during ischemia eventually overcoming Bcl-x_L_-dependent blockade of EndoG release. Furthermore, the link between TUNEL-positive DNA fragmentation and caspase activity, which is true for other cellular models and that has been assumed for cardiomyocytes, was questioned previously by investigations performed in tissue samples [4], [42]. Our data demonstrate that the TUNEL signal is produced by the activity of EndoG without the contribution of caspases in ischemic cardiomyocytes.

In summary, our results provide evidence that 1) EndoG and Bnip3 are expressed abundantly in the differentiated myocardium compared to the embryo, in contrast to the caspase-dependent machinery, 2) EndoG is the link between Bnip3-mediated mitochondrial dysfunction and DNA degradation during ischemia in cardiomyocytes, assembling a protein network unrelated to caspases in postmitotic cells, 3) TUNEL labeling is due to EndoG and is unrelated to caspase activity in ischemic myocytes in vitro and 4) Bcl-x_L_ can prevent transiently caspase-independent DNA fragmentation by blocking EndoG translocation from mitochondria in the presence of Bnip3. All this agrees with a type of caspase-independent cell damage that can take place after the global expression silencing of genes of the caspase-dependent signaling cascade in postmitotic cells such as cardiomyocytes.

## Materials and Methods

### Ethic statement

The investigation with experimental animals conforms to the Guide for the Care and Use of Laboratory Animals published by the US National Institutes of Health (NIH Publication No. 85-23, revised 1996) and the National Guidelines for the regulation of the use of experimental laboratory animals from the Generalitat de Catalunya and the Government of Spain (article 33.a 214/1997) and was evaluated and approved by the Experimental Animal Ethic Committee of the University of Lleida (CEEA) (Permit numbers for dissection of adult rat tissues: CEEA 04-03/08 and for the sacrifice of zero to five-day-old (P0–P5) neonatal rats to obtain tissues and primary cell culture of cardiomyocytes: CEEA 06-03/08).

### Tissues, cardiomyocyte isolation, cell culture and ischemia treatment

Adult three-month-old male Sprague Dawley rats were sacrificed by exposure to carbon dioxide and tissues were dissected as previously described [2]. P0–P5 Sprague–Dawley rat neonates were sacrificed, following the above Guide approved by the CEEA, by decapitation and cardiomyocytes were obtained from the hearts as described elsewhere [43]. Purity of cardiomyocyte cultures was routinely checked by immunocytochemistry with a cardiac sarcomeric α-actinin antibody (clone EA-53, SIGMA, Saint Louis, MO, USA) and was found to be higher than 90% after 24–48 hours *in vitro*. Experimental ischemia of the cultured cells was achieved by culturing cells in Tyrode's solution (NaCl 137 mM, KCl 2.7 mM, Na_2_HPO_4_ 8 mM, KH_2_PO_4_ 1.5 mM, CaCl_2_ 0.9 mM and 0.5 mM, initial pH: 7.2) inside a hypoxic chamber (Ruskin InVivo400, Cultek) continuously monitored for achieving 0.2% O_2_ and 5% CO_2_. Pan-caspase inhibitor Z-Val-Ala-Asp(OMe)-CH_2_F (z-VAD-fmk) (Calbiochem) was added at 100 µM final dilution where indicated.

### Lentiviral vectors for gene silencing and overexpression

EndoG small hairpin RNA interference (shRNA) construct has been described elsewhere [25]. The Bnip3 and Nix shRNA vectors were obtained following the same protocol as for EndoG, using the sequences 5′-GATACCAACAGAGCTGAAATA-3′ and 5′-GAAGACGGGCAAATAATGT-3′, respectively. Viruses were prepared and tittered as described [25]. Treatment of cardiomyocytes was started always five days after transduction, i.e. after seeding. RT-PCR primers for detecting Bnip3 were Forward: 5′-GAATCTGGACGAAGCAGCTC-3′ and Reverse: 5′-AACATTTTCTGGCCGACTTG-3′ with an expected amplicon size of 230 bp, and Nix primers Forward: 5′-CAATGGAGACATGGAGAAGAT-3′ and Reverse 5′-ATGTTTTCGGGTCTACTGGA-3′ produced a 252 bp amplicon. Unr expression was detected to monitor loading as previously reported [25]. All experiments carried out with transduced cardiomyocytes were repeated at least four times. For EndoG-FLAG overexpression we obtained the EndoG-FLAG fragment from the pcDNA3. EndoG-FLAG construct [25] and subcloned it into the pEIGW vector for lentiviral transduction. The open reading frame of human Bcl-x_L_ was amplified from retro-transcribed 293T human cell line total mRNA, was subcloned into the pcDNA3 vector (Invitrogen), sequence-verified and then subcloned into the pEIGW vector for lentiviral-driven overexpression. Viral particles where obtained as previously described [2]. Simultaneous overexpression of EndoG-FLAG and Bcl-x_L_ was achieved by adding equal volumes of viral stocks for each gene at 6 hours *in vitro*, washing and replacing with the required medium 24 hours later. Expression was assessed by Western Blot prior to experimental ischemia, which was performed three days after infection.

### Total and cytosolic protein extraction, Western blot and Immunofluorescence detection

Whole cell lysates and cytosolic protein extracts were obtained and processed as described [25]. Equal amounts of protein were electrophoresed by SDS-PAGE, transferred to PVDF membranes (Amersham), and probed with specific antibodies. Antibodies used were against Apaf-1 (Alexis, ALX-804-349-C100), Bcl-x_L_ (BD Transduction, 601211), Bnip3 (Cell Signaling, 3769), CAD/DFF40 (Millipore AB16926), Caspase-3 (Cell Signaling, 9665), Cytochrome c (Cell Signaling, 4272), EndoG (SIGMA, E5654; ProSci, PSC-3035), FLAG (SIGMA, A2220), Hsp60 (BD Transduction), Prohibitin (NeoMarkers, 292P501F) and all secondary antibodies were from Invitrogen. Commercial antibodies against EndoG were unspecific (band size not matching EndoG size and signal not reduced in cells with efficient specific knockdown of EndoG), we prepared a polyclonal antibody against a peptide from the C-terminal region of EndoG (Antibody Barcelona, BCN4778) useful for Western blot detection but not for immunofluorescence, which was performed after fixing cells either with 4% paraformaldehyde (PFA) for 30 minutes or 100% methanol for 2 minutes, as previously described [25].

### DNA Integrity Assays

Cells were pelleted at the end of each treatment and frozen at −80°C. Pellets from the same experiment were processed at once and processed as previously described [25]. For analysis of DNA high molecular weight degradation, the blocks were then laid into wells of a 1% agarose, 0.5× TBE gel (CHEF grade, SIGMA-Aldrich). Pulse field electrophoresis was performed as previously described [25] in a CHEF DR-II system (BioRad, Hercules, CA, USA) set to the following protocol: run time, 14 h; switch time from 5 to 50 s; voltage gradient, 6 V/cm. For DNA low molecular weight fragmentation, the first volume of DNA extraction obtained after 24 hours of incubation of the agarose blocks [25] was mixed with 100% ethanol at −20°C overnight, centrifuged, washed in 70% ethanol, eluted in TE buffer and electrophoresed in conventional 1.5% agarose gels. Gels were stained with SYBR Safe (Molecular Probes-Invitrogen), visualized by UV exposure and recorded with a Kodak DC290 digital camera. DNA integrity was also assessed by TUNEL staining of cardiomyocyte cultures fixed with 4% PFA after the treatments specified in the figure legend and was performed with the ApopTag kit (CHEMICON) following manufacturer's instructions. Analysis of DNA damage was also performed in cardiomyocyte cultures and cultures of 293T cells (positive control) treated with ischemia in the presence of 100 µM zVAD-fmk diluted in DMSO or an equal volume of DMSO to assess potential effects of the vehicle (Figure S5).

## Supporting Information

Figure S1 **Caspase 3-like enzymatic activity during ischemia in cardiomyocytes and HEK293T cells.** Executioner caspase activity in cultured primary rat neonatal cardiomyocytes exposed to experimental ischemia was measured by detecting the cleavage of the substrate DEVD.AFC as previously reported (25). In brief, the medium of cardiomyocyte cultures was replaced with Tyrode's solution (see Materials and Methods section) and the plates were placed in an hypoxic chamber set at 0.1% Oxygen and 5% CO2 pressures for 0, 4, 6, 12 or 24 hours. The 293T cell line used for preparing the viruses, which express standard amounts of all the regulators of caspase-dependent cell death, was used as a positive control for the assay. Protein extraction from duplicate plates was performed and the protein concentration was measured by the Lowry assay. Equal amounts of protein (25 µg) were incubated at 37°C with the substrate at 50 µM in 96-well plates. Fluorescence produced by cleavage of the substrate was detected with a Bio-tek FL 600 fluorometer (Izasa). Data obtained for different experimental conditions were compared within the linear phase of absorbance increase. Data are mean of three independent experiments performed in duplicates. Bars are s.e.m. *, p<0.01 vs. time 0.
(PDF)

Figure S2 **Sequence position of two different shRNA oligonucleotides for EndoG and Bnip3 knockdown and their effects on ischemia-induced DNAdamage in cardiomyocytes.** a) Two different shRNA sequences were chosen for EndoG (Bahi et al., 2006) and Bnip3 gene knockdown and functionally tested in ischemia-induced DNA damage in cardiomyocytes as described in the Material and Methods section. b) Both shRNA constructs for each gene were equally effective in protecting DNA integrity. Due to the similar effects of both lentiviruses for each gene on DNA integrity protection, only shRNA-1 for each gene was used to conduct the experiments presented in the article.
(PDF)

Figure S3 **Assessment of the specificity of commercial and in-house prepared anti-EndoG antibodies.** For each Western blot, 40 µg of total protein was loaded of neonatal cardiomyocytes transduced with empty viruses, viruses for shRNA-mediated EndoG silencing (shRNA) or viruses for EndoG-FLAG overexpression as well as several cell lines: SH-SY5Y human neuroblastoma, PC12 rat pheochromocytoma, HEK293 human embryonic kidney, HeLa human carcinoma and 3T3 mouse fibroblasts. Antibodies from SIGMA and ProSci (corresponding to the same original product) were used following manufacturer's instructions. In-house produced antibody (AntibodyBcn BCN4778) was used at 1∶3000 dilution. Membranes were developed with Super Signal reagent and images were obtained after exposure of the membranes to Fuji Super RX film. The experiment was repeated several times with similar results. The orange square indicates the region of the EndoG peptide used to immunize rabbits for each antibody.
(PDF)

Figure S4 **Effect of Bcl-xL overexpression in ischemia-induced translocation of EndoG-FLAG.** Immunofluorescence of EndoG-FLAG in control and ischemic (6 hours) cardiomyocytes transduced 3 days before with viruses inducing EndoG-FLAG overexpression or empty viral particles (Ø) in presence or absence of particles inducing Bcl-xL overexpression. Cells were fixed with methanol as described in the Materials and Methods section. This procedure does not allow detection of EndoG-FLAG when released from mitochondria, because of washing out during the procedure, but allows simultaneous detection of the mitochondrial marker Hsp60 and is complementary to the results shown in Figure 5A.
(PDF)

Figure S5 **Ischemia-induced DNA damage in cardiomyocytes and 293T cells in presence of 100 µM zVAD in DMSO or DMSO alone.** Cultures of neonatal rat cardiomyocytes (0.8×10^6^ cells) or confluent cultures of 293T (1×10^6^ cells) were incubated in Tyrode's solution at 0.1% oxygen in a hypoxic chamber during 12 hours in the presence of zVAD-fmk 100 µM (diluted in DMSO) or equal volume of DMSO without caspase inhibitor. Cells were processed as detailed in Materials and Methods section and low molecular weight fragmentation (ladder) was assessed in 1.5% agarose gels. Experiments were repeated twice with identical results.
(PDF)
