# Supplementary figures and images for: EndoG Links Bnip3-Induced Mitochondrial Damage and Caspase-Independent DNA Fragmentation in Ischemic Cardiomyocytes
Source: PLoS One. 2011 Mar 17;6(3):e17998. doi: 10.1371/journal.pone.0017998 (PMC3060094; doi:10.1371/journal.pone.0017998)

# Caspase-3-like activity during experimental ischemia

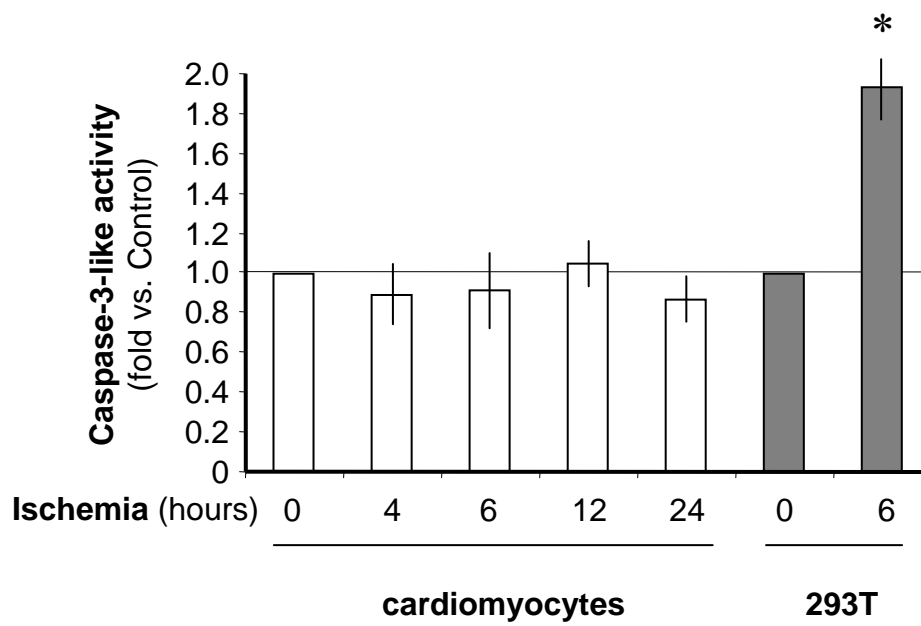

Supplement: Figure S1 — Caspase 3-like enzymatic activity during ischemia in cardiomyocytes and HEK293T cells. Executioner caspase activity in cultured primary rat neonatal cardiomyocytes exposed to experimental ischemia was measured by detecting the cleavage of the substrate DEVD.AFC as previously reported (25). In brief, the medium of cardiomyocyte cultures was replaced with Tyrode's solution (see Materials and Methods section) and the plates were placed in an hypoxic chamber set at 0.1% Oxygen and 5% CO2 pressures for 0, 4, 6, 12 or 24 hours. The 293T cell line used for preparing the viruses, which express standard amounts of all the regulators of caspase-dependent cell death, was used as a positive control for the assay. Protein extraction from duplicate plates was performed and the protein concentration was measured by the Lowry assay. Equal amounts of protein (25 µg) were incubated at 37°C with the substrate at 50 µM in 96-well plates. Fluorescence produced by cleavage of the substrate was detected with a Bio-tek FL 600 fluorometer (Izasa). Data obtained for different experimental conditions were compared within the linear phase of absorbance increase. Data are mean of three independent experiments performed in duplicates. Bars are s.e.m. *, p<0.01 vs. time 0. (PDF) [file pone.0017998.s001.pdf]

## Assessment of ischemia-induced DNA damage

|              | cardiomyocytes |   |   |   | 293T cells |   |   |   |
|--------------|----------------|---|---|---|------------|---|---|---|
| Ischemia     | -              | - | + | + | -          | - | + | + |
| DMSO         | +              | - | + | - | +          | - | + | - |
| zVAD in DMSO | -              | + | - | + | -          | + | - | + |

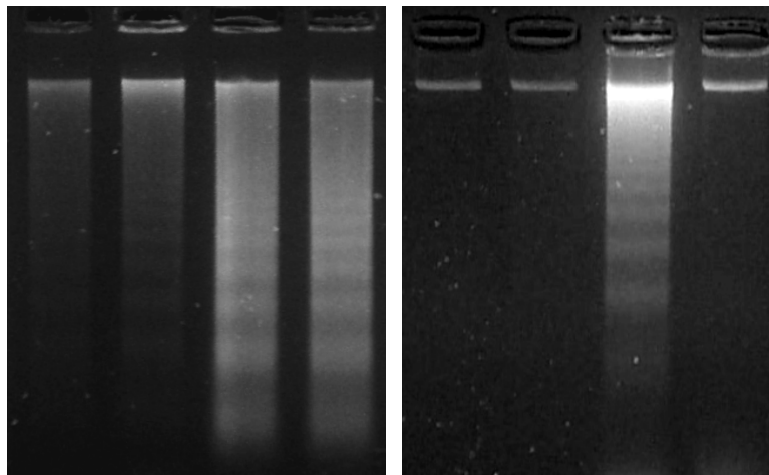

Supplement: Figure S5 — Ischemia-induced DNA damage in cardiomyocytes and 293T cells in presence of 100 µM zVAD in DMSO or DMSO alone. Cultures of neonatal rat cardiomyocytes (0.8×10^6^ cells) or confluent cultures of 293T (1×10^6^ cells) were incubated in Tyrode's solution at 0.1% oxygen in a hypoxic chamber during 12 hours in the presence of zVAD-fmk 100 µM (diluted in DMSO) or equal volume of DMSO without caspase inhibitor. Cells were processed as detailed in Materials and Methods section and low molecular weight fragmentation (ladder) was assessed in 1.5% agarose gels. Experiments were repeated twice with identical results. (PDF) [file pone.0017998.s005.pdf]
