# Supplementary material for: EndoG Links Bnip3-Induced Mitochondrial Damage and Caspase-Independent DNA Fragmentation in Ischemic Cardiomyocytes
Source: PLoS One. 2011 Mar 17;6(3):e17998. doi: 10.1371/journal.pone.0017998 (PMC3060094; doi:10.1371/journal.pone.0017998)

Supplementary Figure 2

Effect on DNA damage of two shRNA constructs for knockdown of EndoG and Bnip3

a

EndoG *Rattus norvegicus* NCBI Reference Sequence: NM_001034938.1
Atgcgcgcgctacgggccggtttgaccctggcgctgggcgccggcctgggcgccgcggcagagcattggcggcggcga
gagggtaaagggccggggctgctgggccgagtgccagtgttgccggttgtcgcggccgatcttcccgcgctgccgggg
ggaccggctggcagcaccggagagttggccaagtacgggctgcccggcgtggcgcagctccggagccgcgagtcctac
gtgctgagctacgacccgcgcacgcgcggtgcgctctgggtgttggagcagctgaggccagagcggctccgtggcgat
ggggaccgtcgcgcctgcgacttccacgaggacgattctgtgcacgcgtaccaccgcgccaccaatgcggactaccgc
ggcagtggctttgaccgcggccacttggcggccgccgccaatcaccgctggagtcagcgggccatggacgacaccttc
tacctgagcaacgtagcgcctcaggtgccacacctcaaccagcatgcct**ggaacaaccttgagaagta**cagcc**g**
cagcttgactcgaacttaccaaaacgtttatgtctgcacagggcctcttttcctgcccaggaccgaggctgatgg
gaagtcctatgtgaagtaccaggttattgggaagaaccacgtggcagtgcccacacacttcttcaaggtgttgatcct
ggaggcagccagtgggcaaatcgagctgcgttcctatgtgatgcccaatgccccggtggatgagactctccctttgga
gcgcttcctagtgcccatcgagagcatcgagcgggcctcgggattgctcttcgtgcccaatattctggctcgagctgg
aaacctcaaggccatcactgctggtagcaagtga

shRNA-1 **shRNA-2**

Bnip3 *Rattus norvegicus* NCBI Reference sequence NM_053420.3
Atgtcgcagagcggggaggagaacctgcagggctcctgggtagaactgcacttcagcaatgggaatgggagcagcgtt
ccagcttccgtctctatttataatggtgacatggaaaaaatactgctggatgcgcagcatgaatctggacgaagcagc
tccaagagctctcactgtgacagcccacctcgctcccagacaccacaa**gataccaacagagctgaaata**gacac
ccacagctttggtgagaaaaacagcactctgtctgaggaagattatattgagagaagaagagaagttgaaagtatcct
gaagaaaaactcagattggatatgggattggtcaagtcggccagaaaatgttcccccaaggagttccttttaaaca
cccgaagcgcacagctactctcagcatga**gaaacacaagcgttatgaa**gaaaggggtattttctcagcagact
ttctgaaggttttccttccatctctgttactgtctcatctgttagccattggattggggatctacattggaaggcgtc
tgacaacttccactag

shRNA-1 **shRNA-2**

b

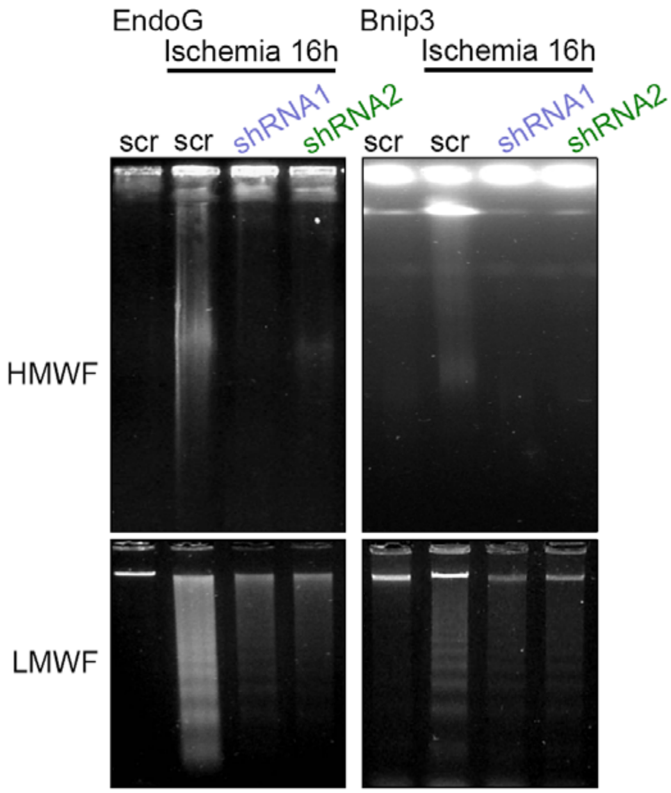

Supplement: Figure S2 — Sequence position of two different shRNA oligonucleotides for EndoG and Bnip3 knockdown and their effects on ischemia-induced DNAdamage in cardiomyocytes. a) Two different shRNA sequences were chosen for EndoG (Bahi et al., 2006) and Bnip3 gene knockdown and functionally tested in ischemia-induced DNA damage in cardiomyocytes as described in the Material and Methods section. b) Both shRNA constructs for each gene were equally effective in protecting DNA integrity. Due to the similar effects of both lentiviruses for each gene on DNA integrity protection, only shRNA-1 for each gene was used to conduct the experiments presented in the article. (PDF) [file pone.0017998.s002.pdf]
