# Supplementary material for: EndoG Links Bnip3-Induced Mitochondrial Damage and Caspase-Independent DNA Fragmentation in Ischemic Cardiomyocytes
Source: PLoS One. 2011 Mar 17;6(3):e17998. doi: 10.1371/journal.pone.0017998 (PMC3060094; doi:10.1371/journal.pone.0017998)

EndoG antibody test

SIGMA, E5654; ProSci, PSC-3035 (correspond to the same antibody sold by different Companies)

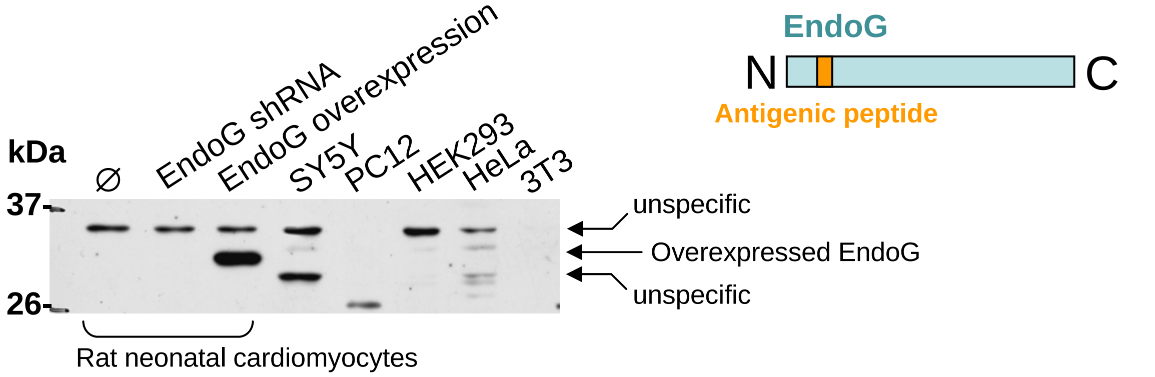

AntibodyBcn BCN4778

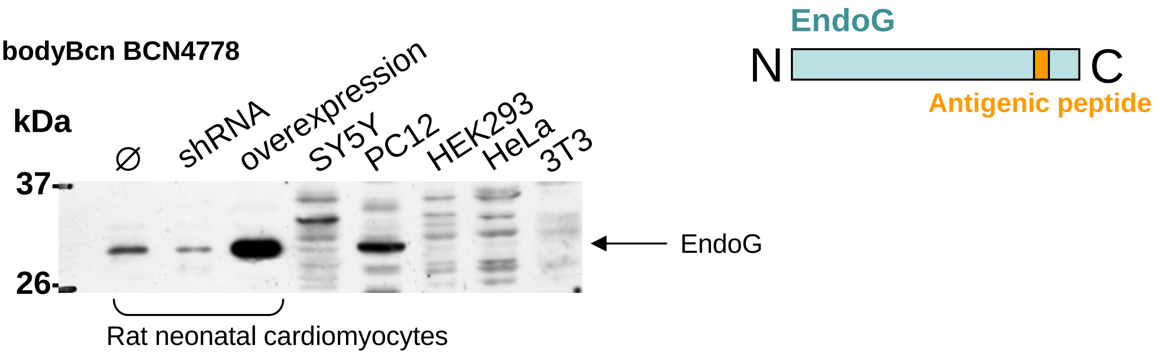

Supplement: Figure S3 — Assessment of the specificity of commercial and in-house prepared anti-EndoG antibodies. For each Western blot, 40 µg of total protein was loaded of neonatal cardiomyocytes transduced with empty viruses, viruses for shRNA-mediated EndoG silencing (shRNA) or viruses for EndoG-FLAG overexpression as well as several cell lines: SH-SY5Y human neuroblastoma, PC12 rat pheochromocytoma, HEK293 human embryonic kidney, HeLa human carcinoma and 3T3 mouse fibroblasts. Antibodies from SIGMA and ProSci (corresponding to the same original product) were used following manufacturer's instructions. In-house produced antibody (AntibodyBcn BCN4778) was used at 1∶3000 dilution. Membranes were developed with Super Signal reagent and images were obtained after exposure of the membranes to Fuji Super RX film. The experiment was repeated several times with similar results. The orange square indicates the region of the EndoG peptide used to immunize rabbits for each antibody. (PDF) [file pone.0017998.s003.pdf]
