# Supplementary material for: EndoG Links Bnip3-Induced Mitochondrial Damage and Caspase-Independent DNA Fragmentation in Ischemic Cardiomyocytes
Source: PLoS One. 2011 Mar 17;6(3):e17998. doi: 10.1371/journal.pone.0017998 (PMC3060094; doi:10.1371/journal.pone.0017998)

EndoG-FLAG mitochondrial localization and effects of Bcl-xL overexpression in ischemia-induced EndoG-FLAG release

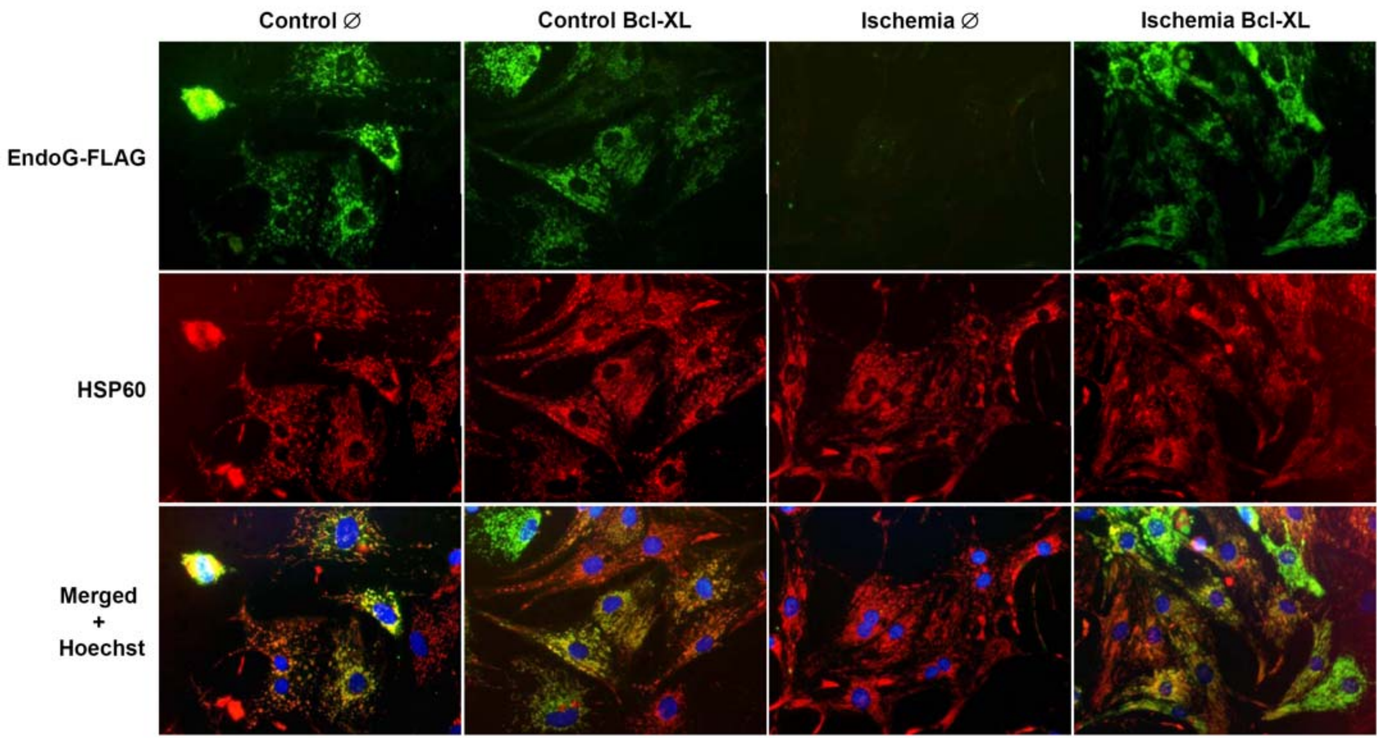

Supplement: Figure S4 — Effect of Bcl-xL overexpression in ischemia-induced translocation of EndoG-FLAG. Immunofluorescence of EndoG-FLAG in control and ischemic (6 hours) cardiomyocytes transduced 3 days before with viruses inducing EndoG-FLAG overexpression or empty viral particles (Ø) in presence or absence of particles inducing Bcl-xL overexpression. Cells were fixed with methanol as described in the Materials and Methods section. This procedure does not allow detection of EndoG-FLAG when released from mitochondria, because of washing out during the procedure, but allows simultaneous detection of the mitochondrial marker Hsp60 and is complementary to the results shown in Figure 5A. (PDF) [file pone.0017998.s004.pdf]
